# An alternative approach to produce versatile retinal organoids with accelerated ganglion cell development

**DOI:** 10.1038/s41598-020-79651-x

**Published:** 2021-01-13

**Authors:** Ellie L. Wagstaff, Anneloor L. M. A. ten Asbroek, Jacoline B. ten Brink, Nomdo M. Jansonius, Arthur A. B. Bergen

**Affiliations:** 1 Department of Clinical Genetics, Amsterdam UMC, University of Amsterdam (UvA), 1105 AZ Amsterdam, The Netherlands; 2 Department of Ophthalmology, University of Groningen, University Medical Center Groningen, 9713 GZ Groningen, The Netherlands; 3 Department of Ophthalmology, Amsterdam UMC, University of Amsterdam (UvA), 1105 AZ Amsterdam, The Netherlands; 4 Netherlands Institute for Neuroscience (NIN-KNAW), 1105 BA Amsterdam, The Netherlands

**Keywords:** Gene expression, Developmental neurogenesis, Stem-cell differentiation

## Abstract

Genetically complex ocular neuropathies, such as glaucoma, are a major cause of visual impairment worldwide. There is a growing need to generate suitable human representative in vitro and in vivo models, as there is no effective treatment available once damage has occured. Retinal organoids are increasingly being used for experimental gene therapy, stem cell replacement therapy and small molecule therapy. There are multiple protocols for the development of retinal organoids available, however, one potential drawback of the current methods is that the organoids can take between 6 weeks and 12 months on average to develop and mature, depending on the specific cell type wanted. Here, we describe and characterise a protocol focused on the generation of retinal ganglion cells within an accelerated four week timeframe without any external small molecules or growth factors. Subsequent long term cultures yield fully differentiated organoids displaying all major retinal cell types. RPE, Horizontal, Amacrine and Photoreceptors cells were generated using external factors to maintain lamination.

## Introduction

Worldwide, an estimated 253 million people are blind or have some form of a visual impairment^1^. Some of the main causes of vision loss are chronic ocular diseases, such as glaucoma and age-related macular degeneration (AMD), which are characterised by a large degree of clinical, genetic and cellular heterogeneity. This makes the development of effective cures and treatments challenging. Many new therapeutic strategies, such as small molecule-, gene- and stem cell-replacement therapies are currently under development. For example, the small molecule Sunitinib has been shown to protect retinal ganglion cells in optic neuropathy models^2^. Also, a potential RPE65 gene therapy for one form of Leber’s congenital amaurosis has recently been approved by the FDA^3^, and an RPE stem cell replacement therapy for macular degeneration is currently in clinical trials^4^. However, therapeutic strategies involving the retina remain an extraordinary challenge, and once lost, vision cannot be restored. Consequently, there is a large need for new treatments that would improve the vision and quality of life of a substantial group of patients.

Since the introduction of human induced pluripotent stem cells (hiPSCs) in 2007^5^, and subsequently human organoids in 2009^6^ research into cell-based regenerative medicine has advanced rapidly. By utilising an extensive range of whole organoid systems including stomach, liver, pancreas, lung, kidney, brain and retina, researchers have modelled many complex diseases such as cancer, cystic fibrosis and microcephaly^7–13^. In preclinical trials, groups have been able to use whole organoid systems, testing experimental drugs and observing any effects on a smaller scale^14^.

To successfully model diseases in a whole system environment, every cell type needs to be present within these organoids, and proper cell development and maturation cannot be rushed. A drawback of organoid research is the large amount of time that is needed to produce these cells in high quantities needed for disease modelling and preclinical pharmacological testing. The retina contains on average 3.5 million pigmented epithelial cells^15^, 1 million ganglion cells^16^, 117 million rods and 6.5 million cones^17^, all present in a highly organised multi-layered structure, with support cells also present.

The generation of retinal organoids can be divided into three stages. Embryoid body formation is the first stage of in vitro retinal organogenesis, in which dissociated ESCs/iPSCs differentiate into free-floating embryoid bodies (EBs). Secondly, neural enrichment takes place, whereby whole EBs are plated and allowed to grow out their epithelial-fate cells, while forming neuron enriched centres. Finally, organoids develop by mechanically lifting off the neural centres and allowing them to fold and form in a free-floating environment into spherical organoids.

Culture methods and protocols vary between groups, but a common theme is a similar order of successive developmental stages along the retinal lineage, specified by specific cell marker expression. In general, immature retinal pigmented epithelial cell markers are expressed first, around day 35 of differentiation, while mature markers are not found until after 50–60 days of differentiation. Mature ganglion cells have been found to appear after around 40 to 50 days of culturing, and photoreceptors expressing immature and mature markers around day 70 and day 100 respectively^18,19^. When using these 3D floating culture techniques, RPE cells also need a further 4 weeks to mature and create the monolayer that is observed in vivo*.*

Zhu and coworkers were previously able to reduce these prolonged iPSC-RPE culture and differentiation times by using a 3D matrigel culture model, initiating a direct differentiation toward RPE cells^20^. Matrigel is a gel with a relatively defined rich mixture of extracellular matrix proteins and growth factors such as collagen, laminin and epidermal growth factor (EGF). It is commonly used in stem cell culturing as a coating material to promote in vitro cell growth and contribute to structural organisation. Zhu et al. encased stem cell clumps in a 3D matrigel droplet, letting them develop into embryoid bodies with positive early retinal lineage markers present after 5 days of differentiation. After the dissociation of the embryoid bodies and subsequent replating of cells onto transwell membranes, early pigmentation could be seen by day 18, with a mature RPE monolayer containing tight ZO-1 positive junctions seen by day 30. This method of forming retinal cysts has since been successfully repeated^21^, forming an early pigmentation monolayer in roughly 20 days; a significantly shorter and more reproducible timeframe when compared to other methods of generating RPE (Table 1).

**Table 1:** A comparison of popular retinal differentiation protocols against our MG/FF combination protocol, showing the expression timeline of specific cell markers used to confirm the generation of general retinal organoids, retinal ganglion cells (RGCs), retinal pigmented epithelium (RPE) and photoreceptors (PR).

Cell focus	Method			Method adapted from	IHC confirmation		PCR Confirmation		References	Year
	Stage	Culture	Days		Marker	Day	Marker	Day		
General	SFEBq			Nakano et al.	PAX6	24			^50^	2015
	Embryoid bodies	Floating	0–18		CHX10	24				
	Neurospheres	Floating	18+		MITF	24 (low)				
					PKCa	24				
					BRN3	50				
					Crx	50				
					NRL	150				
General	Embryoid bodies	Floating	0-6	Keller et al.	PAX6	10	PAX6	6	^54^	2009
	Rosettes	Adherent	6–16		RAX	10	RAX	6		
	Neurospheres	Floating	16+		CHX10	30	MITF	16		
					MITF	30	CHX10	23		
					ZO-1	30	RPE65	40		
							CRX	60		
							OPSIN	70		
General	Embryoid bodies	Floating	0-6	Meyer et al.	PAX6	20	PAX6	20	^57^	2011
	Rosettes	Adherent	6-16		CHX10	20–25	BIII	20		
	Neurospheres	Floating	16+		ISL1	20–25	BRN3	30		
	Seperation of Retinal spheres	Floating	20-25		ZO-1	40	CRX	30		
					MITF	40	MITF	40		
					BRN3	80	RPE65	40		
					PKCa	80	BEST1	40		
					CRX	80	NRL	60		
					RCVRN	80				
					NRL	80				
General	SFEBq				PAX6	20	PAX6	10	^46^	2009
	Embryoid Bodies	Floating	0-21		RAX	20	MITF	10		
	Neurospheres	Floating	21+		CHX10	20	RAX	20		
	PRs	Adherent	90		MITF	35	CHX10	20		
					ZO-1	100	RPE65	120		
					RHO	140				
General	Embryoid Bodies	Adherent	0-28		PAX6	14	LHX2	14	^48^	2017
	Neurospheres	Floating	28+		RAX	14	MITF	14		
					LHX2	14	RAX	14		
					MITF	14	PAX6	14		
					CHX10	14	VSX2	14		
					BRN3	21	CRX	14		
					CRX	21	BRN3	21		
					RCVRN	42	RCVRN	35		
General	SFEBq			Nakano et al.	RAX	7	VSX2	10	^58^	2016
	Embryoid Bodies	Floating	0-18		PAX6	7	RAX	10		
	Neurospheres	Floating	18-41		LHX2	7	ISL1	10		
					MITF	10	ATOH7	10		
					VSX2	10	BRN3	10		
					BRN3	41	RCVRN	10		
							CRX	15		
							PAX6	15		
							NRL	18		
General	Embryoid Bodies	Matrigel	0-4	Ohlemacher et al. and Zhu et al.	RAX	4			This study	2019
	Rosettes	Adherent	4-11		PAX6	4				
	Neurospheres	Floating	11+		SOX2	4				
					ISL1	28				
					HuC/D	28				
					PKCa	28				
					ZO-1	56				
					RCVRN	91				
					GFAP	96				
					RBPMS	96				
					RHO	168				
General	Embryoid Bodies	Floating	0-7	Meyer et al.	PAX6	8	PAX6	4	^27^	2014
	Rosettes	Adherent	7-28		RAX	12	LHX2	4		
	Neurospheres	Floating	28+		MITF	14	RAX	8		
					VSX2	14				
					HuC/D	35				
					BRN3	35				
					RCVRN	63				
					RHO	119				
General/PR	SFEBq				PAX6	18			^18^	2012
	Embryoid Bodies	Floating	0-18		CHX10	18				
	Neurospheres	Floating	18-126		MITF	18				
					PKCa	18				
					BRN3	30				
					RCVRN	43				
					NRL	126				
					RHO	126				
RGC	Embryoid Bodies	Floating	0-4		HuC/D	25	RAX	3	^60^	2016
	RGCs	Adherent	4-30		BIII	25	ISL1	3		
					BRN3a	45	ATOH7	3		
							PAX6	3		
							BRN3B	3		
							MITF	8		
							RPE65	11		
RGC	Embryoid Bodies	Floating	0-4		MATH5	20	RAX	4	^28^	2018
	Rosettes	Adherent	4-8		BRN3B	40	LHX2	4		
	RGCs	Adherent	8-40		ISL1	40	CHX10	12		
							MATH5	12		
							CRX	12 (low)		
							MITF	12		
							BRN3B	19		
RGC	Rosettes	Adherent	0-14		RAX	14			^51^	2014
	Neurospheres	Floating	14-40		PAX6	14				
					ZO-1	14				
					BRN3	40				
RGC	SFEBq			Nakano et al.	RAX	24	RAX	6	^49^	2015
	Neurospheres	Floating	0-26/29		PAX6	24	PAX6	6		
	RGCs	Adherent	26/29+		MATH5	34	CRX	6		
					BRN3	34	CHX10	6		
					CRX	34	PKCa	18		
							MITF	18		
							BRN3	24		
							MATH5	24		
RGC/RPE	Embryoid bodies	Floating	0–7	Meyer et al.	PAX6	20	PAX6	20	^19^	2015
	Rosettes	Adherent	7-16		CHX10	30	RAX	20		
	Neurospheres	Floating	16+		BRN3	40	CHX10	30		
	Seperation of Retinal spheres	Floating	20-25		RCVRN	50	BRN3	40		
					HuC/D	70	ISL1	50		
					ISL1	70	HuC/D	50		
		Floating			RBPMS	70				
RPE	Embryoid bodies	Floating	0–7		Pax6	13			^53^	2014
	Rosettes	Adherent	7–16		MITF	13				
	Neurospheres	Floating	16+		VSX2	15				
					ZO-1	60				
RPE	Embryoid Bodies	Matrigel	0-5		PAX6	3	PAX6	5	^20^	2013
	RPE	Adherent	5-25		ZO-1	1 (EB)	RAX	5		
					RAX	5	CHX10	5		
					CHX10	15	MITF	10		
PR/RPE	Embryoid Bodies	Floating	0-30		Rho	45	PAX6	15	^47^	2012
	Neurospheres	Adherent	30-60		OPN1SW	45	RPE65	30		
					RPE65	45	CHX10	45		
					ZO-1	45	CRX	60		
							RCVRN	60		
PR	SFEBq			Nakano et al.	PAX6	37			^56^	2015
	Embryoid Bodies	Floating	0-12		BRN3b	37				
	Neurospheres	Floating	12-90		CRX	37				
					RCVRN	37				
					PKCa	67				
					Arr	67				
					NRL	67				
					OPN1SW	90				
PR	Embryoid Bodies	Floating	0-6	Meyer et al.	PAX6	56	RAX	56	^55^	2018
	Neurospheres	Adherent	6+		RAX	56	VSX2	56		
					RCVRN	56	PAX6	56		
					CRX	56	RCVRN	56		
					BRN3B	140	CRX	56		
							MITF	56		
PR	SFEBq			Nakano et al.	BRN3	35	OPN1SW	12	^36^	2017
	Embryoid Bodies	Floating	0-10/12		RCVRN	45	VSX2	30		
	Neurospheres	Floating	10/12+		RHO	160	RCVRN	30		
							CRX	65		
							NRL	100		
							ARR3	100		

**Figure 1:**
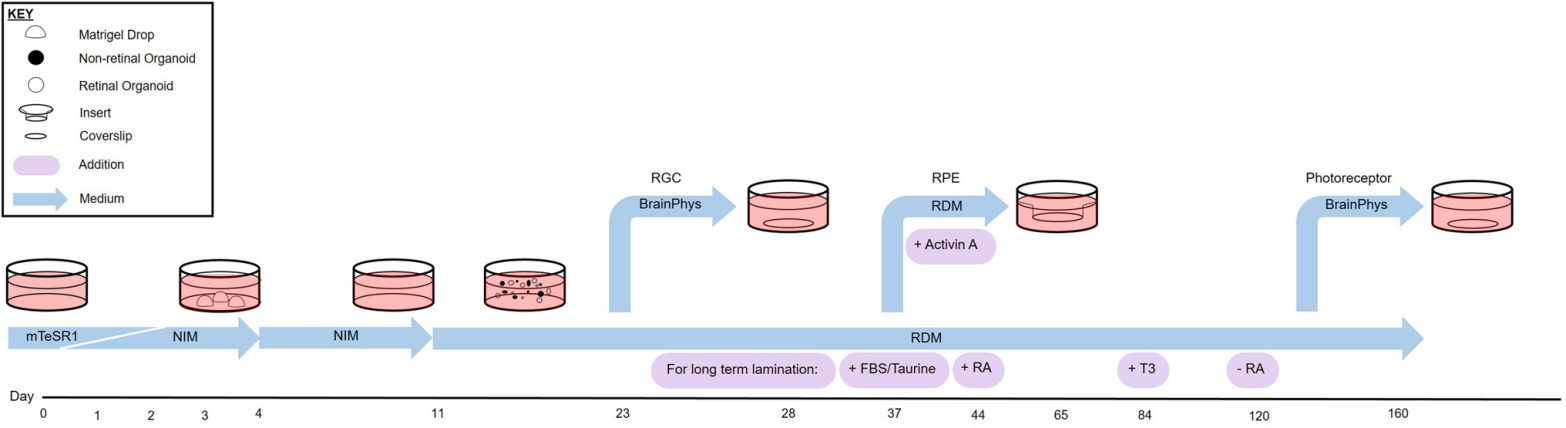
From left to right: schematic diagram of the generation of retinal organoids over time. Initially, human stem cell clumps are cultured in a 3D environment by suspension in solidified matrigel drops (MG). During the next 4 days incubation, with medium changing gradually from stem cell maintenance medium (mTeSR1) to neural induction medium (NIM), organised embryoid bodies are formed. These are subsequently plated in an adhesive culture (2D) environment allowing the outgrowth of epithelial cells for a further 7 days to enrich the neural centres. These neural centres are scraped off on day 11 and cultured 3D in a floating suspension environment (FF) using retinal differentiation medium (RDM), where they form early retinal organoids by day 14. Organoids are kept in this environment until required, depending on the desired cell type being produced. To develop and maintain long term lamination, FBS and Taurine are added from day 37. After a week, Retinoic Acid (RA) is also added. At day 84, Triiodo-l-Thyronine (T3) is also added and Retinoic Acid concentrations are halved. By day 120, Retinoic Acid is removed completely to allow for rod and cone maturation, with FBS, Taurine and T3 still present. For retinal ganglion and photoreceptor cells, organoids are dissociated and plated on coated coverslips in complete BrainPhys medium to stimulate neuronal outgrowth at days 23 and 160 respectively. For retinal pigmented epithelium, organoids at developmental day 37 are dissociated and seeded on growth factor reduced matrigel coated inserts, where they are grown into a pigmented monolayer for 4 weeks, supplemented with Activin A.

In this study, we combine the well-established free-floating culture protocol, referred to herein as FF (free-floating)^19^ with the 3D Matrigel culture method^20^, referred to herein as MG (Matrigel), an overview of which can be found in Fig. 1. This creates retinal organoids in a shorter timeframe compared to previously published methods and, consequently, this method could drastically reduce culture time for relevant experimental therapeutic studies.

## Results

### Encasing stem cells in matrigel yields morphologically more mature and highly structured embryoid bodies

**Figure 2:**
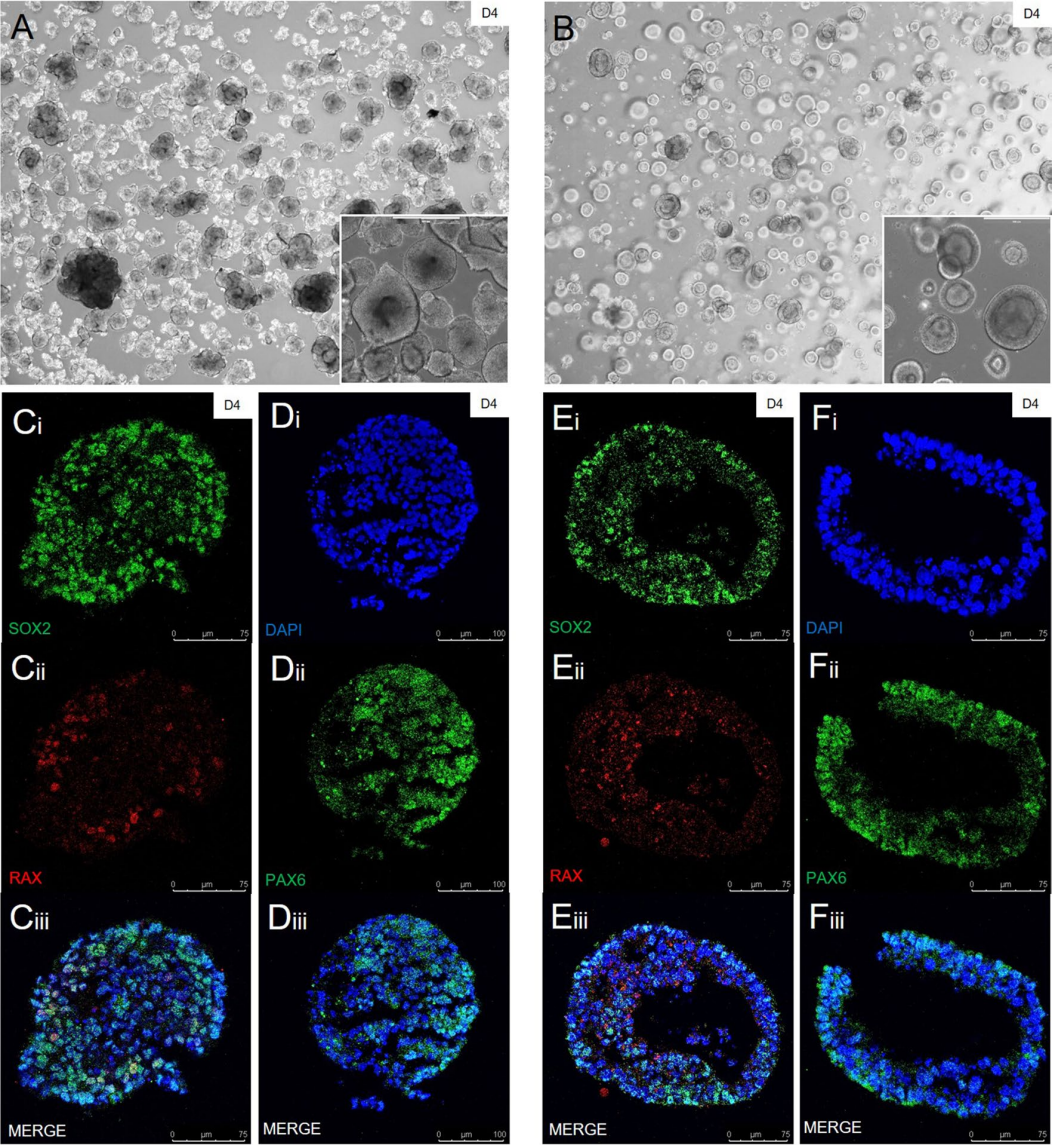
Differences between EBs grown in matrigel embedment (MG) culture vs floating suspension (FF) culture. (**A**,**B**) Under a light microscope, EBs grown using a floating culture technique (**A**) look morphologically unorganised when compared to EBs grown using matrigel 3D embedment culture technique (**B**) by day 4. Highly reproducible, this morphological difference (detailed in (**A**,**B**) inserts, scale bar = 400 µm) is clear to see throughout entire cultures. This is also evident when looking at cross sections using IHC. (**C**–**F**) Floating cultures generate a mass of cells, whereas the EBs cultured in matrigel drops possess a clear stratified layer of cells with a hollow centre, much like an already matured organoid, while also staying within the neural lineage. The acquisition of neural identity was confirmed in both the floating culture derived bodies (**Ci-iii**,**Di-iii**), as well as the matrigel derived bodies (**Ei-iii**,**Fi-iii**) using common neuronal markers SOX2 and PAX6 (both in green). The general retinal marker RAX (in red) was also confirmed in both sets of EBs.

The first stage of generating retinal organoids is to grow embryoid bodies (EBs). EBs are predominantly generated by breaking up stem cell clones into around 100 µm clusters and growing them in a 3D floating culture environment to generate spheres that contain a multitude of different early progenitor cells (FF). However, in our hands, this generally resulted in relatively unstructured and disorganised looking EBs (Fig. 2a). In contrast, our MG EBs encapsulated in a 3D matrigel drop looked, in general, much more structured and mature, almost like fully grown organoids rather than early stage EBs (Fig. 2b). In order to confirm that our EBs were more organised at this early stage without losing any neural potential, we performed IHC staining of EBs with the well-known early neural and retinal developmental markers SOX2, PAX6 and RAX. Comparisons between floating (Fig. 2c,d) and matrigel (Fig. 2e,f) derived EBs shows that similar amounts of SOX2, PAX6 and RAX are expressed in both methods. The results show that EBs grown encased in a 3D matrigel environment are more structurally organised when compared to their floating counterparts, without compromising their neural identity.

### Generation of retinal organoids: timely expression of key retinal developmental markers throughout development

**Figure 3:**
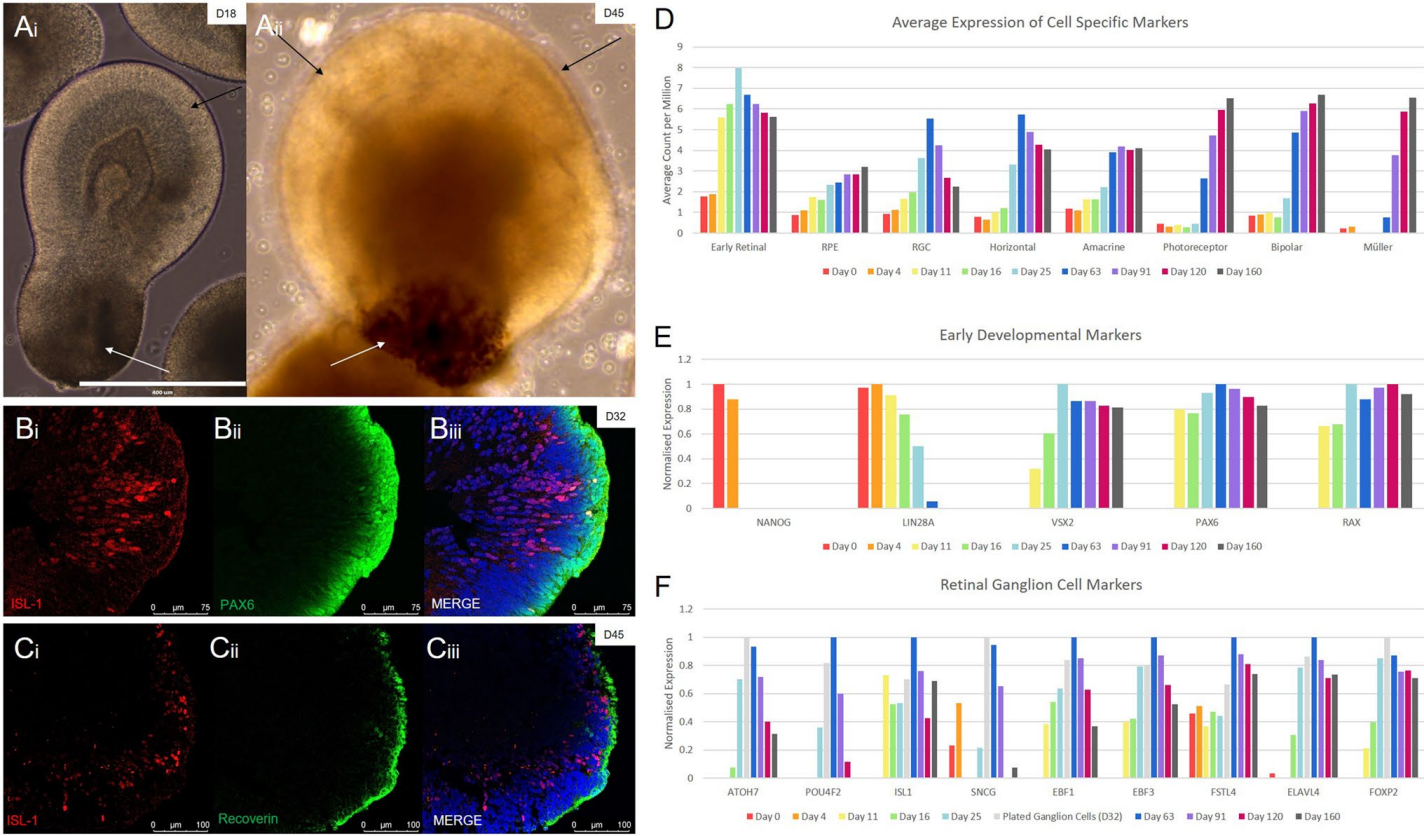
Confirmation of retinal organoid development. (**Ai-ii**) Whole organoids were observed to have clear portions of neural retina (black arrows) and pigmented cells (white arrows) as early as day 18 (Scale Bar = 400 µm). (**Bi-iii**) Cross sectioned IHC analysis showed the formation of a distinct ganglion cell layer, shown here using the ganglion marker ISL1 (in red). (**Ci-iii**) A separation of the inner ganglion layer (shown by ISL-1 in red), and the outer photoreceptor layer (shown by the common photoreceptor progenitor marker RCVRN, in green), was seen by day 45. (**D**–**F**) Quantitative RNA sequencing data of cell specific markers showed sequential development of retinal cell types in our organoids in line with other published protocols. (**D**) An average of multiple markers for each cell type show cell development over time. (**E**) Common pluripotent and early developmental markers showed expression with comparable results to previously published protocols (Table 1). (**F**) Retinal ganglion cell markers show peak expression between Day 32 and 63 before decreasing over time. Ganglion subtype markers were also shown to be present (FOXP2, FSTL4), as well as markers strongly associated with ISL1 and SNCG (EBF1, EBF3).

To generate retinal organoids, we modified a previously published FF organoid protocol^19^. Initially, we started out with a direct matrigel based differentiation method that we previously used to generate stem cell derived RPE monolayers^21^. Next, we investigated the effects of combining this initial 3D matrigel culture into a retinal organoid protocol that was subsequently merged with, and followed up by, conventional floating methods (MG/FF). We observed a more complex and organised morphology of the EBs in our MG/FF protocol compared to solely FF protocols, resulting in morphologically distinct early stage organoids within 14 days. Microscopic inspection of MG/FF retinal organoids showed that both pigmented and neural sections are present (Fig. 3a).

Fully differentiated retinal organoids characteristically have a laminated morphology, with ganglion cells found on the inner-most layer of the organoid, and photoreceptors located on the outside. To confirm this in our system, we sectioned and stained our MG/FF organoids at different developmental timepoints. By 32 days, a defined layer of ganglion cells has developed within the organoids, similar to an in vivo retina, that stain positive for ISL-1 and PAX6 (Fig. 3b). We observed a large difference between the strong PAX6 staining on the outer edge of the organoid and weak PAX6 staining in the inner layer (Fig. 3b). Close up analysis of the inner layer of ganglion cells show strong co-expression of both ISL-1 and PAX6 at day 32 (Supplementary Fig. S1). This advanced by day 45 to include the outer layer of photoreceptor progenitors (Fig. 3c). However, we observed that when using our MG/FF protocol, the organoids ultimately start to lose this structure around day 50–60 of differentiation. This could be offset by the addition of external factors such as Taurine, Retinoic Acid and Triiodo-l-Thyronine (T3) alongside FBS, a mixture commonly used to enhance long term layering and photoreceptor development^22^.

In our MG/FF culture, the progression of the organoids through the different lineages was measured by the presence of different retinal developmental markers. Using RNA sequencing we analysed the presence and expression of multiple different cell type specific markers at a number of different timepoints. Figure 3d shows the average trend in expression for different sets of cell specific markers. We found that early retinal markers (n = 7) increase dramatically once differentiation is forced into the retinal lineage, before peaking at an early retinal organoid timepoint at day 25. RPE-specific markers (n = 5) increase steadily over the course of the differentiation, whilst RGC (n = 22) and horizontal cell (n = 7) specific markers increased in expression during the earlier cultures, reaching their peak at day 63 and subsiding during long term cultures. Amacrine markers (n = 8) also increase in expression during the early cultures before reaching a consistent level of expression from day 63 onwards. Finally, photoreceptor (n = 32), bipolar (n = 6) and Müller (n = 2) cell markers are not present during the earlier timepoints, but increase rapidly from day 63 onwards until the final timepoint at day 160.

We analysed a few commonly used markers in detail. We found high RNA expression of the known pluripotency markers NANOG and LIN28 in the initial stem cell and embryoid body stages, before decreasing once retinal differentiation started. Overlapping with the expression of these markers, the early neural and retinal progenitor markers PAX6 and RAX are expressed from day 11 onwards. Highly similar to the RAX expression pattern, the optic cup specific marker VSX2 increases from a low expression on Day 11 to a peak in expression on Day 25, remaining highly expressed throughout the culture. The ganglion specific markers ATOH7 and POU4F2 start to be expressed at day 16 and 25 respectively, peaking at days 32 and 63, before gradually decreasing in the later timepoints up until day 160. The presence of ganglion cells was also suggested by the expression of other commonly used markers such as ISL1, SNCG and HuC/D (In this case ELAVL4 refers to HuD), all increasing in expression during the early stages of organoid development, before peaking between days 32 and 63, and decreasing gradually up to day 160.

Interestingly, we also found expression of RGC subtype specific markers, such as FOXP2 and FSTL4. These are markers of F-type ganglion cells and ON/OFF ganglion cells respectively, and were also highly expressed between days 32–63. There was also expression of genes known to interact with ganglion markers ISL1 and SNCG^23^. These genes (EBF1, EBF3), although not confirmed as specific markers for RGCs, are a good indicator that RGC-related genes are interacting (Fig. 3f).

### Presence of RPE, inner retinal cell types and photoreceptors within MM/FF derived organoids

#### Development of the RPE

**Figure 4:**
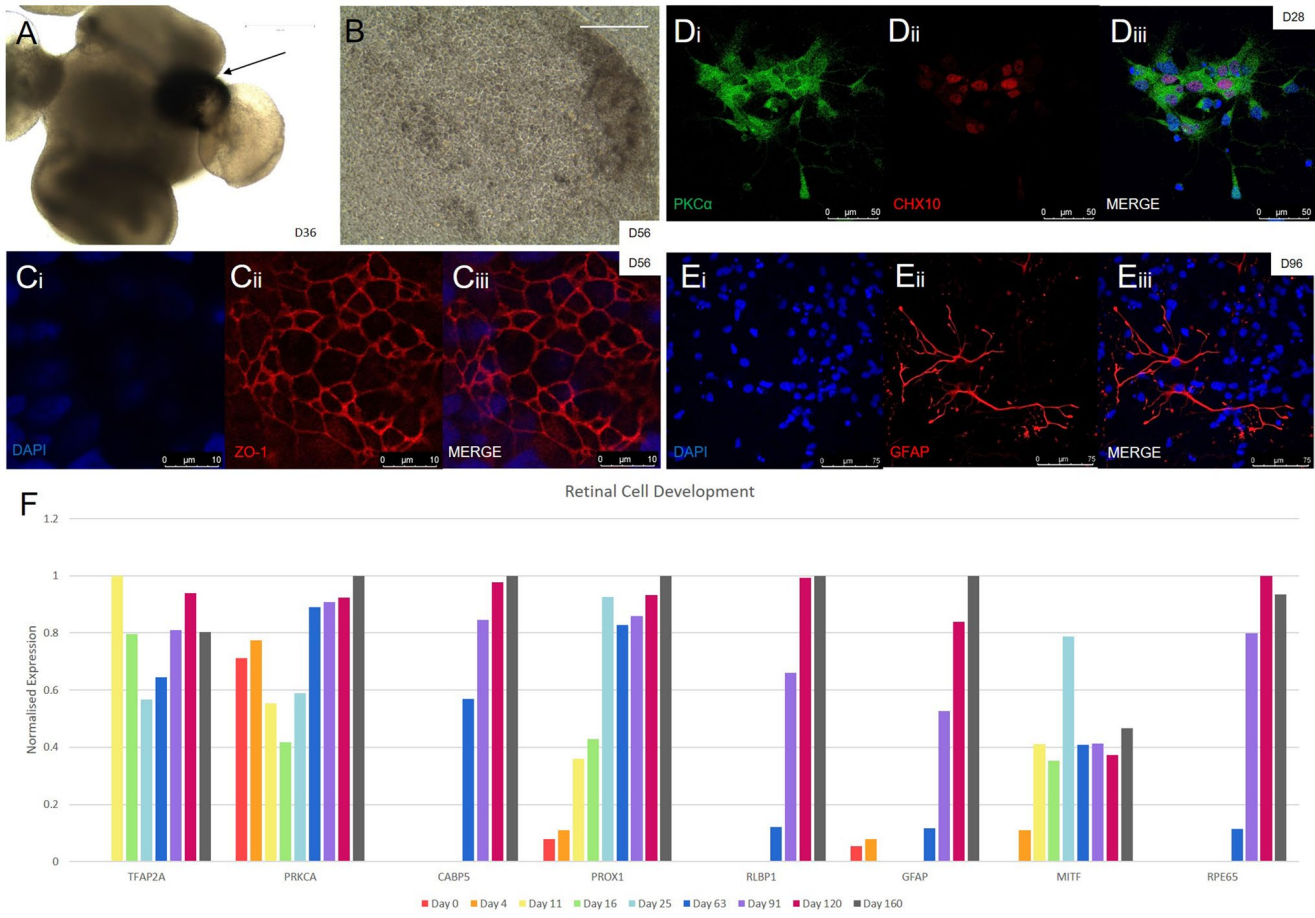
Generation of RPE and other retinal cell types. (**A**) A subset of organoids developed visible pigmented areas by day 36. (**B**,**Ci-iii**) These were dissociated and grown as a monolayer that had the characteristic cobblestone appearance and ZO-1 tight junction presence (in red) of RPE. (**Di-iii**) According to our quantitative marker data, bipolar cells appeared in culture within 4 weeks. The presence of bipolar cells was confirmed by staining for PKCα (in green) at day 28. (**Ei-iii**) Dissociated organoids grew out Müller cells, which were confirmed by staining for GFAP (in red). (**F**) In addition to bipolar, horizontal, Müller and pigmented epithelial cells, amacrine cells were also found within the organoid, suggested by RNA sequencing data.

Although our focus was to differentiate early ganglion cells for glaucoma disease modelling, we also aim to use our organoids for a variety of different disease models. Therefore we further analysed the cultures for the presence of RPE and other retinal cells. We observed areas of pigmentation within our organoids by 25 days of total differentiation (Fig. 4a). Pigmented organoids were subsequently dissociated into single cells and seeded on transwell inserts, to grow out and mature into an RPE monolayer (Fig. 4b) that strongly expresses the tight junction protein ZO-1, (Fig. 4c). RNA analysis of whole organoids indicates early expression of RPE progenitors such as MITF, which is present from day 4 onwards with the highest expression at day 25, before making way for the mature RPE marker RPE65, that appears from day 63 onwards (Fig. 4f).

#### Horizontal, bipolar, and Müller cells

The horizontal marker PROX1 increases in expression gradually over the differentiation, with a significant increase between days 16 and 25, sustaining high expression after day 25 until the final timepoint at day 160. Similarly, the amacrine marker GAD1 exhibits low levels of expression until day 63 onwards. Bipolar cell markers PKCa and CaBP5 are expressed in early and late stage cultures respectively, with confirmation using IHC at day 28 (Fig. 4d). Müller cell markers GFAP and RLBP1 show no expression in the early timepoints, but steadily increase in expression from day 63 until the final timepoint at day 160, confirmed at day 96 by IHC staining with GFAP (Fig. 4e).

#### Photoreceptors

**Figure 5:**
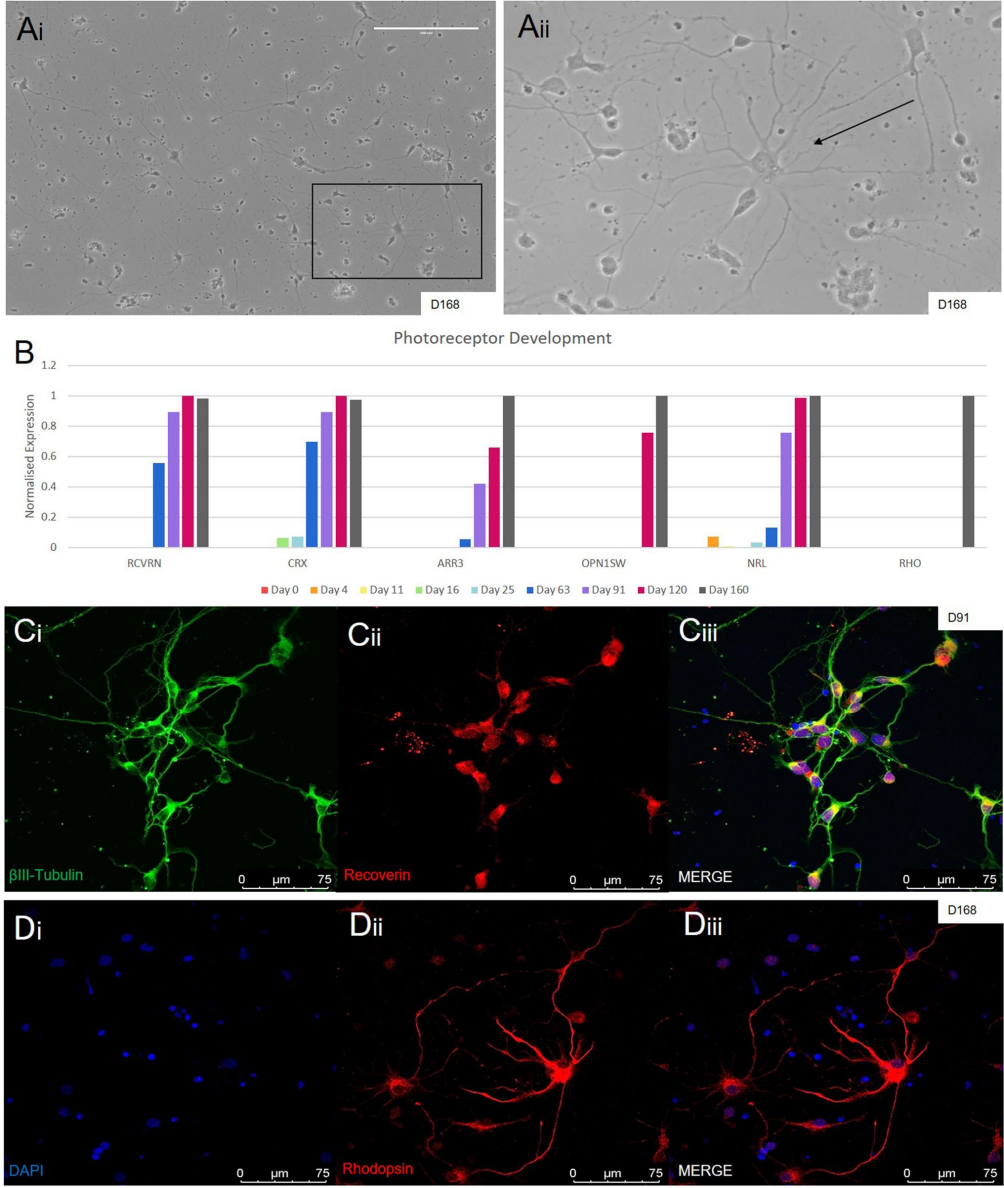
Generation of photoreceptors. (**Ai-ii**) Widefield images of plated photoreceptors at D168 (black arrow), obtained from dissociated organoids, show outgrowth that is morphologically different to plated ganglion cells (Shown in Fig. 6a) (Scale bar = 200 µm). (**B**) Quantitative RNA sequencing data of common photoreceptor progenitor markers CRX and RCVRN showed increasing expression after day 63, while the mature cone and rod markers ARR3, OPN1SW and NRL, RHO also increased over time, although not as dramatically. (**Ci-iii**) Expression of photoreceptor progenitor markers was seen with IHC staining of RCVRN (in red) with the general neuronal marker β-III Tubulin (in green). (**Di-iii**) More mature photoreceptors were stained using anti-rhodopsin antibody ROD (in red).

Generally, the last major retinal cell type to develop is the photoreceptor^24^, which is also true using our MG/FF protocol. Older organoids dissociated at day 160 showed growth of individual photoreceptors (Fig. 5a, black arrow) that are morphologically clearly distinguishable from plated ganglion cells. Upon RNA quantification we observe the photoreceptor progenitor markers RCVRN and CRX expressed from day 63 onward, and significantly increasing over time (Fig. 5b). This is also confirmed by IHC using RCVRN and βIII-Tubulin antibodies (Fig. 5c). The mature cone-specific (ARR3, OPN1SW) and rod-specific (NRL, RHO) photoreceptor markers show high expression from day 91 onwards. From this timepoint onwards, organoid morphology does not show specific laminated layers of cells. However, individual plated mature photoreceptors were confirmed using IHC staining of Rhodopsin, a commonly used photoreceptor marker (Fig. 5d) that has previously shown expression within the cell body of early photoreceptors^25^.

### Long-term organoid lamination

We initially observed that in our matrigel based protocol, withholding additional external factors meant that long term lamination was lost around day 50–60 onwards.

**Figure 6:**
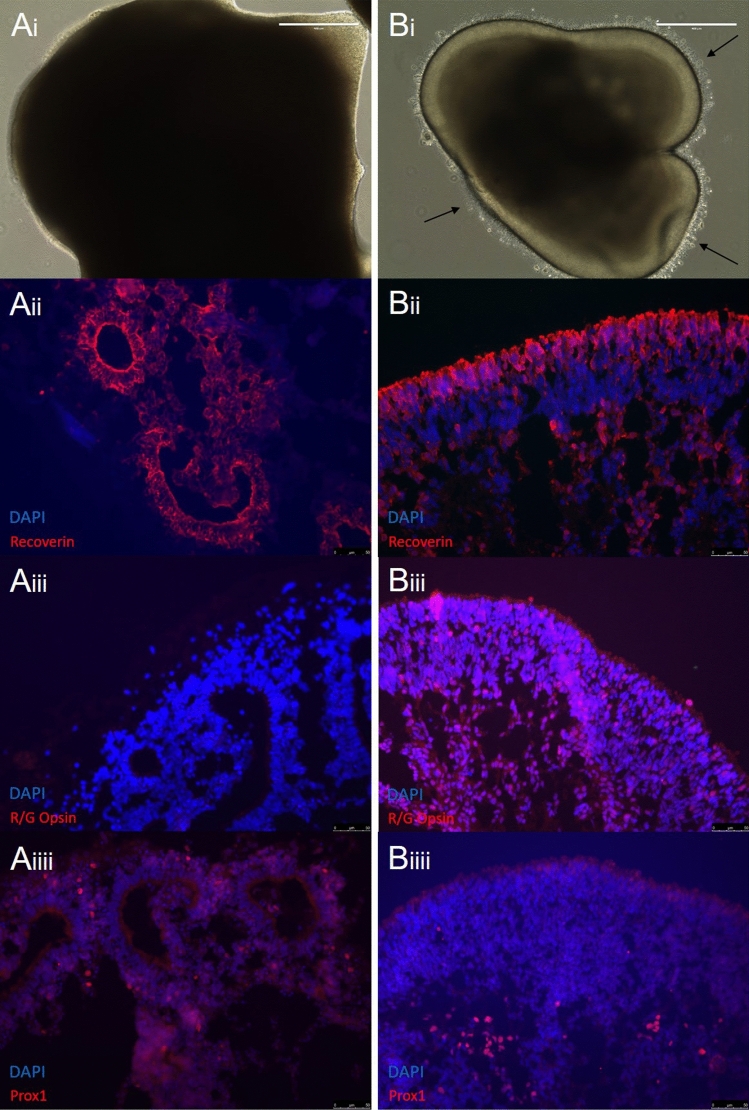
Long term lamination of retinal organoids. Organoids were either ‘treated’ by the addition of the external factors FBS, Taurine, RA or T3, or left ‘untreated’ in standard RDM. A comparison between long term cultures of untreated (**Ai**–**Aiii**) and treated (**Bi**–**Biii**) retinal organoids showed significant differences. (**Ai**) Untreated organoids appear extremely dark and dense, and contain photoreceptor rosettes. (**Bi**) Treated organoids retain their golden outer layer, and develop photoreceptors outer segments (black arrows) (Scale bars = 400 µm). (**Aii**) Photoreceptors develop within defined rosettes on the outermost part of the untreated organoid, shown by staining with the photoreceptor progenitor marker, RCVRN (in red). (**Bii**) Treated organoids contain an outer layer of photoreceptors surrounding the organoid, shown by RCVRN (in red). (**Aiii**) Untreated organoids also do not develop Red-Green cones by Day 160, suggested by lack of RNA expression and IHC staining. (**Biii**) The addition of T3 in the treated organoid cultures stimulated the development of Red-Green cone photoreceptors. (**Aiiii**) Although having lost the lamination of the organoid and developing rosettes, untreated organoids did still develop other cells around the rosettes, such as horizontal cells, found at the bottom of the photoreceptors. (**Biiii**) Treated organoids retain an multi-layered appearance, with photoreceptors on the outer edge of the organoid, and other cells positioned below as found in the in vivo retina.

However, all cell types still develop within the organoid, while the morphology of the organoid turns into a sphere of rosettes. Within these rosettes photoreceptors develop and mature with other cell types appearing at the outer edges of the rosettes, in their corresponding layers. To rectify this loss of lamination, we added a mixture of FBS, Taurine, Retinoic Acid and T3, commonly used to enhance long term layering and photoreceptor development. Initially we copied a timeframe derived from previously published protocols, starting treatment around day 45^26,27^. However, this hardly improved the morphological outcome (data not shown). Apparently the crucial cellular changes have already started before this timepoint, due to the quicker development induced by the 3D matrigel start of our protocol. We adjusted our timing, and found that adding the factors at day 37 results in retinal organoids that keep their distinct golden halo appearance and layering past day 160. From around day 90–100, the beginnings of photoreceptor outer segments can be seen, and by day 160, these cumulate in a distinct layer around the golden halo areas of the organoid (Fig. 6b). IHC stainings of organoids without or with treatment starting at day 37 show significant differences in layering and rosette formation. Untreated control organoids had an outer ring of rosettes, where photoreceptors developed (Fig. 6a). Organoids treated from day 37 show a distinct lamination of cells with photoreceptors present in the outer layer, followed by horizontal cells further in (Fig. 6b).

### Outgrowth of RGCs

**Figure 7:**
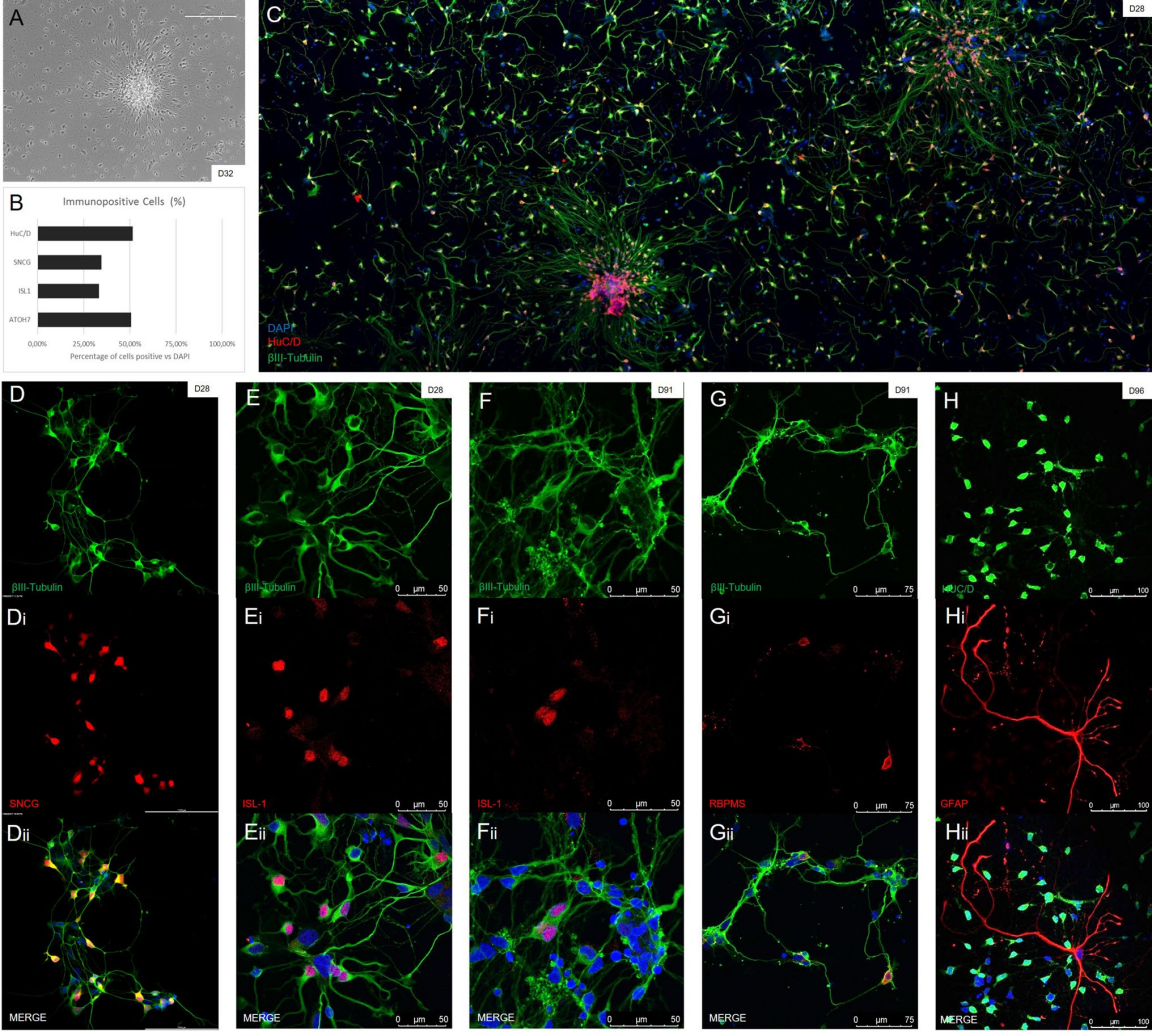
Confirmation of the presence of retinal ganglion cells. (**A**) Widefield images of ganglion cells observed in culture showed polar cell bodies and an outgrowth of long axons by 32 days of culture. (**B**) When calculating the efficiency of ganglion cell generation, we found approximately 30–50% of cells were positive for ganglion cell specific markers when compared to general DAPI staining. The early ganglion marker ATOH7 showed positive staining in over 50% of cells (n = 510), with SNCG, a marker highly present in mature RGCs alongside BRN3B, showing positive staining in approximately a third of cells (n = 1779). Other commonly used RGC markers, although not completely specific, showed similar levels of positive staining. ISL1 was present in approximately a third of total cells (n = 1251), with HuC/D staining observed in almost half of all cells (n = 746). (**C**) After dissociating organoids at day 23, we observed a consistent amount of RGC outgrowth after 5 days. (**Di-iii**, **Ei-iii**) IHC staining of early RGC cultures using multiple ganglion markers SNCG and ISL-1 (both in red) showed positive expression and neurite outgrowth within 4 weeks of differentiation, shown by βIII-Tubulin (in green). (**Fi-iii**, **Gi-iii**) Plated cells from timepoints after 90 days of culturing show expression of the ganglion specific markers ISL-1 and RBPMS (both in red) co-stained with the general nerual marker βIII-tubulin (in green). (**Hi-iii**): Later stage cultures at day 96 showed expression of the RGC and amacrine marker HuC/D (in green) alongside Müller cells, identified using GFAP (in red).

An important drawback of generating retinal ganglion cells for cell replacement treatments is the length of time needed to generate these cells and the great quantities that are needed. Most recently published protocols need between 40 and 50 days to generate retinal ganglion cells (see Lee et al.^28^). A majority of these add a number of different external factors or molecules that modulate important developmental pathways, and forcibly promote differentiation into the ganglion cell lineage. Our MG/FF protocol enables us to generate ganglion cells within 28 days (Fig. 7a), which was confirmed by stainings with multiple ganglion markers (Fig. 7d–h). MG/FF organoids dissociated and plated at day 28 show significant percentages of retinal ganglion cells. Quantification shows dissociated cultures have a yield of over 50% ATOH7 positve cells (n = 510), a well known marker for RGCs. Additionally, SNCG, a marker highly expressed in mature human RGCs alongside BRN3B^29^, shows a yield of over 30% positive cells (n = 1779). Other commonly used RGC markers ISL1 and HuC/D have yields of over 30% (n = 1251) and 50% (n = 746) respectively (Fig. 7b) These plated cells show healthy outgrowth stained with βIII-Tubulin, as shown in Fig. 7c. Long term culturing of ganglion cells is another problem for cell replacement therapies. However we can still achieve outgrowth of ganglion cells after 90 days, with positive ISL1 and RBPMS (Fig. 7f–g) as well as HuC/D (Fig. 7h) expression.

## Discussion

The generation of a rapid, reliable human model system to study retinal diseases has long been a challenging limitation in eye research. In the past, the most common methods were either to use immortal cell lines such as ARPE-19 or explants from freshly harvested human donor eyes. Neither of these are ideal, either due to different expression profiles for immortal cells, or short lifespans for explants. The generation of murine organoids in 2011^13^, and human retinal organoids a year later^18^, was huge leap forward in the field of retinal research. However, a drawback of organoid research remains the lengths of time that are needed for proper maturation and cell development. Therefore, the generation of protocols that reduce developmental time, such as described in this study, is beneficial.

The addition of specific inhibiting or promoting factors can direct retinal differentiation in vitro through the manipulation of certain signalling pathways. The Wnt, BMP, TGF-β and insulin signalling pathways are all responsible for sending developing organoids down the retinal lineage^30^. It has been shown previously that IGF-1 plays an important role in the formation of neural retina^31^ and the prolonged development of retinal ganglion cells^32^. Furthermore, in animal models, it has been observed that insulin receptors are abundantly expressed in adult rat RGCs^33^. In fact, injections of insulin have been shown to promote dendrite regeneration in murine models^34^. Some of these inhibiting or promoting factors are present in the undefined matrigel matrix, thereby affecting the early stages of embryoid body formation in our protocol.

By using matrigel, we introduced an average concentration of IGF-1 of more than 1.5× the concentration used in previous cultures^32^ (Supplemental Table S2, provided by Corning https://www.corning.com/emea/en.html) at an early developmental stage. We added this high concentration to our FF embryoid bodies to see if this would also benefit the culture, however the expression levels of early developmental markers were no different to standard floating cultures over the embryoid body phase (Data not shown). Interestingly, embedding cells in a 3D-matrix creates a hypoxic microenvironment when compared to free-floating cultures^35^, that has been shown to lead to improved retinal cell yield^36–39^. Expression of IGF1 has also been found to be upregulated in hypoxic cells when compared to normoxic cells^40,41^ further increasing this effect. Other important growth factors found in matrigel, such as TGF-β, can influence different signalling pathways, such as the BMP and TGF-β signalling pathways. Many protocols try to influence these pathways by adding external factors, including IGF-1, Noggin, Forskolin, DKK-1, FGF2 and others to promote or inhibit different signalling pathways^37,42–44^. One example is the insulin signalling pathway that plays an essential role in neurogenesis and the development and differentiation of retinal tissue^45^. A large boost of key undefined growth factors present in matrigel at an early developmental stage could explain the defined appearance that we observe in our early EBs.

We used a range of specific cell type markers to compare our results with those of previously published studies (Table 1). The initial expression of certain developmental markers differed extensively across the many previously published studies. However in most cases, the early developmental markers PAX6 and RAX are simultaneously expressed first in culture, before early retinal-specific markers such as CHX10 and MITF join them. Our data shows PAX6, RAX, CHX10 and MITF expression by day 11, having slightly earlier expression than comparable studies^19,46,47^. The initial expression of retinal cell-specific markers varies greatly between organoid protocol publications (Table 1), but roughly follows the same temporal order. In general, ganglion cells are among the first cells to fully differentiate and mature, with RPE cells appearing somewhere in the middle, other cell types inbetween, and photoreceptors being the last. With a couple of exceptions, the expression profile and appearance of other cell types found in the retinal organoid culture system are usually not documented. We found robust RNA expression of multiple markers for all cell types found within the retina.

In our study, retinal ganglion cells were present from an early point, with RNA sequencing showing robust ISL1 expression from day 11, ATOH7 expression from day 16 and the mature marker POU4F2 appearing by day 25, similar to data presented in other studies^48,49^. This was further confirmed by way of IHC, where ATOH7, SNCG, ISL1 and HuC/D positive cells were present significantly quicker than a large number of previously published protocols^27,28,50^. Our cultures showed a high yield of ganglion cells, between roughly 30–50% depending on the marker, which is comparable to or better than similar studies of 22%, 10% and 45% in 40 days^28,51,52^, but at an earlier timepoint. Although we found no evidence that an earlier development of retinal ganglion cells could lead to an earlier death, further work should be completed to determine the viability and longevity of the cells through long term culturing. For modelling diseases such as Glaucoma or Axenfeld–Rieger Syndrome, our protocol can generate good yields of ganglion cells within 4 weeks, without adding any external factors. However, this compromises the organoid lamination. By adding FBS, Taurine, Retinoic Acid and T3, long term layering can be achieved.

Apart from ganglion cells, we also generated and identified other retinal cell types in our organoids, such as RPE cells. Robust expression of early RPE markers such as MITF was shown within 11 days, and an RPE monolayer with tight junctions was confirmed by day 56, which mirrored other studies focused around generating RPE cells^47,53^. Our retinal organoids expressed not only the conventional markers for RGCs and RPE that are commonly used for confirmation, but markers for bipolar cells, Müller glia cells, horizontal cells and amacrine cells were also present and confirmed by IHC or RNA sequencing. Photoreceptors were the last cells to appear as expected, and although they did not appear to develop before other (photoreceptor-focused) studies, the expression and localisation staining was comparable. Early markers such as RCVRN and CRX were seen by day 63, which is a known time for photoreceptor progenitors to appear^54–56^, and this was followed by the more mature markers, with IHC staining of photoreceptors by Rhodopsin confirming the maturation, as shown previously^25,36,57^. When we do not add factors to our organoids to improve lamination, we find a lack of expression of the medium and long wave opsins. However with the addition of T3, a known promotor of red-green cone development^22^, IHC staining indeed showed the presence of medium and long wave opsin.

Our findings suggest that the addition of high doses of growth factors, such as IGF-1, in matrigel during the early stages of development may boost temporal retinal organoid differentiation compared to conventional floating body protocols^27,28,58^. This leads to outgrowth of RGCs within 4 weeks and layered organoids that develop all cell types found within the retina. Although the effect we observe from the matrigel is most pronounced in the accelerated generation of retinal ganglion cells, there is robust expression of other gene markers early on in development. However relying on gene expression alone is not advised, and therefore it is recommended for other cell types, that further work should be continued to look at both the cell yield and localisation of the cells within the organoid.

We refrained from adding specific cell-promoting external factors such as Retinoic Acid or Taurine into the medium, which led to loss of accelerated development, lack of lamination and rosette formation over time. Nonetheless, we observed photoreceptors in comparable timeframes to previous studies^27,36,46,47,54^. Adding commonly used factors to promote lamination in long term organoid cultures also worked in our organoid differentiation protocol, and results in photoreceptor maturation and outer segment formation. Interestingly, we had to add these factors earlier compared to other published protocols otherwise the proper layering was also lost. This emphasises the importance of timing in development, and confirms that organoids generated with Matrigel encased EBs undergo accelerated differentiation.

## Conclusions

Overall, our study presents a rapid and reproducible protocol to generate versatile retinal organoids for disease modelling in a shorter timeframe. An initial boost using a 3D matrigel culture containing high concentrations of growth factors leads to early differentiation of EBs. These present with a more structured and organised morphology compared to floating culture EBs, and result in a shorter timeframe for generation of retinal ganglion cells. Due to the lack of additional growth factors or small molecules in the organoid culture, we provide a versatile culture method that can be used to reliably model and study a number of retinal diseases. Common additions over time can be used to produce and maintain organoid lamination and develop fully differentiated organoids, including photoreceptor outer segments.

## Materials and methods

### Organoid formation

The H1 hESCs (WiCell) were maintained on hESC-qualified matrigel (Corning) coated 6 well plates with mTeSR1 medium (STEMCELL Technologies), passaging twice a week using gentle cell dissociation reagent (STEMCELL Technologies). Organoid formation followed the rough timeline and stage specification published previously^19^, with some major alterations. An overview of our method, referred to herein as MG/FF (combination method) is presented in Fig. 1.

Briefly, stem cell clones were dissociated into small clumps (around 100 µm in diameter) using 0.5 mM EDTA in PBS, and then suspended in undiluted matrigel (Corning). The matrigel suspension was then plated with 8 × 25 µl individual drops per 35 mm well, totally encasing the stem cell clumps in a matrigel microenvironment. After gelling for 10 min at 37 °C, 3 ml per well was added of a 3:1 ratio of mTeSR1 and neural induction medium (NIM), consisting of DMEM/F12 (-L-Glutamine) (1:1), N2 supplement, non-essential amino acids, heparin (2 µg/ml), PenStrep and GlutaMAX. Day of matrigel encasement was annotated as day 0, with the medium being changed on day 1, (1:1 ratio), day 2 (1:3 ratio) and day 3 (full NIM), as shown previously^19^. After four days of differentiation, all of the stem cell clumps had formed into large organised embryoid bodies (EBs) (Fig. 2b), that were taken out of the 3D matrigel droplets with cell recovery solution (Corning). The whole EBs were then plated on 6 well plates coated with hESC-qualified Matrigel (Corning), that were incubated for a further 7 days, with NIM media changes every other day. On day 11, the neural centres were carefully dislodged and transferred to a 60 mm dish containing retinal differentiation medium (RDM), which consisted of DMEM/F12 (-l-Glutamine) (3:1), B27 supplement, non-essential amino acids, PenStrep and GlutaMAX. Retinal organoids were observed by day 14, and kept in culture until the desired time with RDM media changes every other day. For long term layering experiments, treatment started on day 37. At day 37, RDM was supplemented with 10% FBS (Thermofisher) and 100 µM Taurine (Sigma). After 7 days, 1 µM of Retinoic Acid (RA) (Sigma) was also added to the RDM. The medium was changed every 2–3 days until day 84. At day 84, RA concentration was halved and 40 ng/µl T3 (Sigma) was added. Medium was changed with FBS, Taurine, RA and T3 every 2–3 days until day 120. At day 120, RA was removed completely to allow for photoreceptor maturation. All other additions stayed in the medium until the end of experiment.

To generate individual neural retinal cell types, whole organoids were dissociated using StemPro Accutase (Thermofisher) following the previously published protocol^19^, and plated on poly-Ornithine and Laminin double coated glass coverslips with BrainPhys medium (STEMCELL Technologies), which was changed every other day for up to 10 days after plating. For retinal pigmented epithelial (RPE) cell generation, organoids were dissociated and seeded on growth factor reduced matrigel coated 24-well inserts (both Corning) at a density of 1.0 × 10^6^ cells for up to 5 weeks in RDM with Activin-A (Agrenvec). To passage RPE cells, monolayers were dissociated using Accutase (Thermofisher) for 20 min, before being counted using the Countess automated cell counter (Thermofisher), and seeded at the same density as previously.

### Immunohistochemistry

For whole organoid immunohistochemistry (IHC) analysis, EBs and organoids were fixed for 25 and 40 min respectively using 4% Paraformaldehyde (PFA) in PBS, before being mounted in O.C.T Tissue-Tek (Sakura Finetek) on dry ice, and sectioned using a microtome at 10 µm thickness. For dissociated organoids, cells were fixed in 4% PFA for 20 min. Fixation was followed by 3 washes with PBS for 5 min before blocking for an hour at room temperature with 10% donkey serum and 0.2% Triton X-100 in PBS for permeabilisation. Primary antibodies (Supplementary Table S1) were applied overnight in 0.1% Triton X-100, 5% donkey serum in PBS at 4 °C. Following 3 washes of 5 min with PBS, the secondary antibodies were applied for an hour at room temperature in 0.1% Triton X-100, 5% donkey serum mix and DAPI. After washes with PBS and water slides were dried for an hour at room temperature in the dark. Coverslips were mounted onto slides using ProLong Gold Antifade Mountant (Thermofisher).

### Quantitative RNA sequencing (RNAseq)

RNA sequencing was performed as previously described^59^. Briefly, for each time-point, 5 organoids were lysed in Trizol and RNA was extracted. The quality of the resulting RNA was assessed on a BioAnalyzer. For the RNAseq library preparations, the KAPA mRNA HyperPrep kit was used according to manufacturer’s instructions with 50 ng input RNA for each sample. Libraries were single-end sequenced using the Illumina HiSeq 8000 platform. Gene expression was shown as an average count per million across all genes for specific cell types for a general overview of expression. For detailed expression comparison, the highest value of expression for each gene was arbitrarily set at 1, with expression values of other timepoints shown relative to the highest expression per gene.

## Supplementary Information

Supplementary Information.

## Data Availability

All data generated or analysed during this study are included in this published article (and its Supplementary Information files).
