# Supplementary material for: An alternative approach to produce versatile retinal organoids with accelerated ganglion cell development
Source: Sci Rep. 2021 Jan 13;11:1101. doi: 10.1038/s41598-020-79651-x (PMC7806597; doi:10.1038/s41598-020-79651-x)
Supplement: Supplementary file 1 — Supplementary Information. [file 41598_2020_79651_MOESM1_ESM.docx]

An alternative approach to produce versatile retinal organoids with accelerated ganglion cell development

Ellie L. Wagstaff^1^, Anneloor L.M.A. ten Asbroek^1^, Jacoline B. ten Brink^1^, Nomdo M. Jansonius^2^, Arthur A.B. Bergen^1,3,4^*

1. Department of Clinical Genetics, Amsterdam UMC, Univ(ersity) of Amsterdam, Neurosciences and Reproduction & Development, Meibergdreef 9, Amsterdam, Netherlands (NL)
2. Department of Ophthalmology, University of Groningen, University Medical Center Groningen, Groningen, NL
3. Department of Ophthalmology – Amsterdam UMC, Univ(ersity) of Amsterdam, Meibergdreef 9, Amsterdam, NL
4. Netherlands Institute for Neuroscience (NIN-KNAW), Amsterdam, NL

* All correspondence to be sent to: aabergen@amsterdamumc.nl

Supplementary Material

Table 1 – Antibodies

| Antibody | Dilution | Company | Order Number |
| --- | --- | --- | --- |
| Chx10 | 1:100 | Millipore | AB9016 |
| HuC/D | 1:200 | Thermofisher | A21271 |
| Islet-1 | 1:200 | DSHB | 40.2D6 |
| Pax6 | 1:250 | Genetex | GTX113241 |
| Rax | 1:200 | Santa Cruz | sc-271889 |
| PKCα | 1:50 | BD Biosciences | 610107 |
| Recoverin | 1:500 | Millipore | AB5585 |
| Rhodopsin | 1:100 | Millipore | MAB5356 |
| ZO-1 | 1:200 | Santa Cruz | sc-8146 |
| RBPMS | 1:500 | PhosphoSolutions | 1830-RBPMS |
| GFAP | 1:200 | Dako | Z0334 |
| Sox2 | 1:200 | Abcam | Ab75627 |
| βIII-Tubulin | 1:400 | Millipore | AB15708A4 |
| Prox1 | 1:1000 | Merck | AB5475 |
| R/G Opsin | 1:200 | Merck | AB5405 |
| ATOH7 | 1:100 | Novus Bio | NBP1-88639 |
| SNCG | 1:500 | Abnova | H00006623-M01 |

Table 2 – Matrigel components

Taken from <https://www.corning.com/media/worldwide/cls/documents/CLS-DL-CC-026%20DL.pdf>

| Amounts of Growth Factors (GF) Present in Corning Matrigel Matrix vs. Growth Factor Reduced (GFR) Corning Matrigel Matrix | | | |
| --- | --- | --- | --- |
| Growth Factor | Range of GF Concentration in Corning Matrigel Matrix | Average GF Concentration in Corning Matrigel Matrix | Typical GF Concentration in GFR Corning Matrigel Matrix |
| EGF | 0.5-1.3 ng/mL | 0.7 ng/mL | < 0.5 ng/mL |
| bFGF | < 0.1-0.2 pg/mL | n.a.* | n.d.** |
| NGF | < 0.2 ng/mL | n.a.* | < 0.2 ng/mL |
| PDGF | 5-48 pg/mL | 12 pg/mL | < 5 pg/mL |
| IGF-1 | 11-24 ng/mL | 16 ng/mL | 5 ng/mL |
| TGF-β | 1.7-4.7 ng/mL | 2.3 ng/mL | 1.7 ng/mL |
| *n.a.- not applicable | | | |
| **n.d.- not determined | | | |

Table 3 – Genes used in overview of expression found in Figure 3D

| Cell Type | Early Retinal | RPE | RGC | Horizontal | Amacrine | Photoreceptor | Bipolar | Müller |
| --- | --- | --- | --- | --- | --- | --- | --- | --- |
| Gene | VSX2 | TRPM3 | ATOH7 | PROX1 | TFAP2A | RCVRN | PRKCA | RLBP1 |
|  | LHX2 | MITF | POU4F2 | GAD1 | CALB2 | CRX | CABP5 | GFAP |
|  | PAX6 | BEST1 | ISL1 | ONECUT2 | STX1A | RHO | CAMK2B |  |
|  | RAX | RPE65 | SNCG | CHAT | ASCL1 | NRL | GRM6 |  |
|  | SFRP2 | RRH | PRPH | NTRK1 | C1QL2 | ARR3 | KCNG4 |  |
|  | TUBB3 |  | FOXP2 | ONECUT1 | EPHA8 | OPN1SW | SCN3A |  |
|  | FGF19 |  | LDB3 | ONECUT3 | TACR1 | KCNV2 |  |  |
|  |  |  | GAP43 |  |  | EPHA10 |  |  |
|  |  |  | EBF3 |  |  | GUCA1A |  |  |
|  |  |  | EBF1 |  |  | GUCA1B |  |  |
|  |  |  | CHRNB3 |  |  | CNGB1 |  |  |
|  |  |  | NEFM |  |  | GNGT1 |  |  |
|  |  |  | SLC17A6 |  |  | PDE6A |  |  |
|  |  |  | NEFL |  |  | PDE6H |  |  |
|  |  |  | TFAP2D |  |  | PDE6C |  |  |
|  |  |  | CDH6 |  |  | THRB |  |  |
|  |  |  | TRHR |  |  | PDC |  |  |
|  |  |  | CNTN2 |  |  | ROM1 |  |  |
|  |  |  | BARHL2 |  |  | AIPL1 |  |  |
|  |  |  | RUNX1 |  |  | USH2A |  |  |
|  |  |  | TBR1 |  |  | GNAT2 |  |  |
|  |  |  | NTF3 |  |  | GNAT1 |  |  |
|  |  |  |  |  |  | NR2E3 |  |  |
|  |  |  |  |  |  | SAG |  |  |
|  |  |  |  |  |  | RXRG |  |  |
|  |  |  |  |  |  | LHX4 |  |  |
|  |  |  |  |  |  | RBP3 |  |  |
|  |  |  |  |  |  | RGR |  |  |
|  |  |  |  |  |  | EYS |  |  |
|  |  |  |  |  |  | PPEF2 |  |  |
|  |  |  |  |  |  | ABCA4 |  |  |
|  |  |  |  |  |  | RPGRIP1 |  |  |


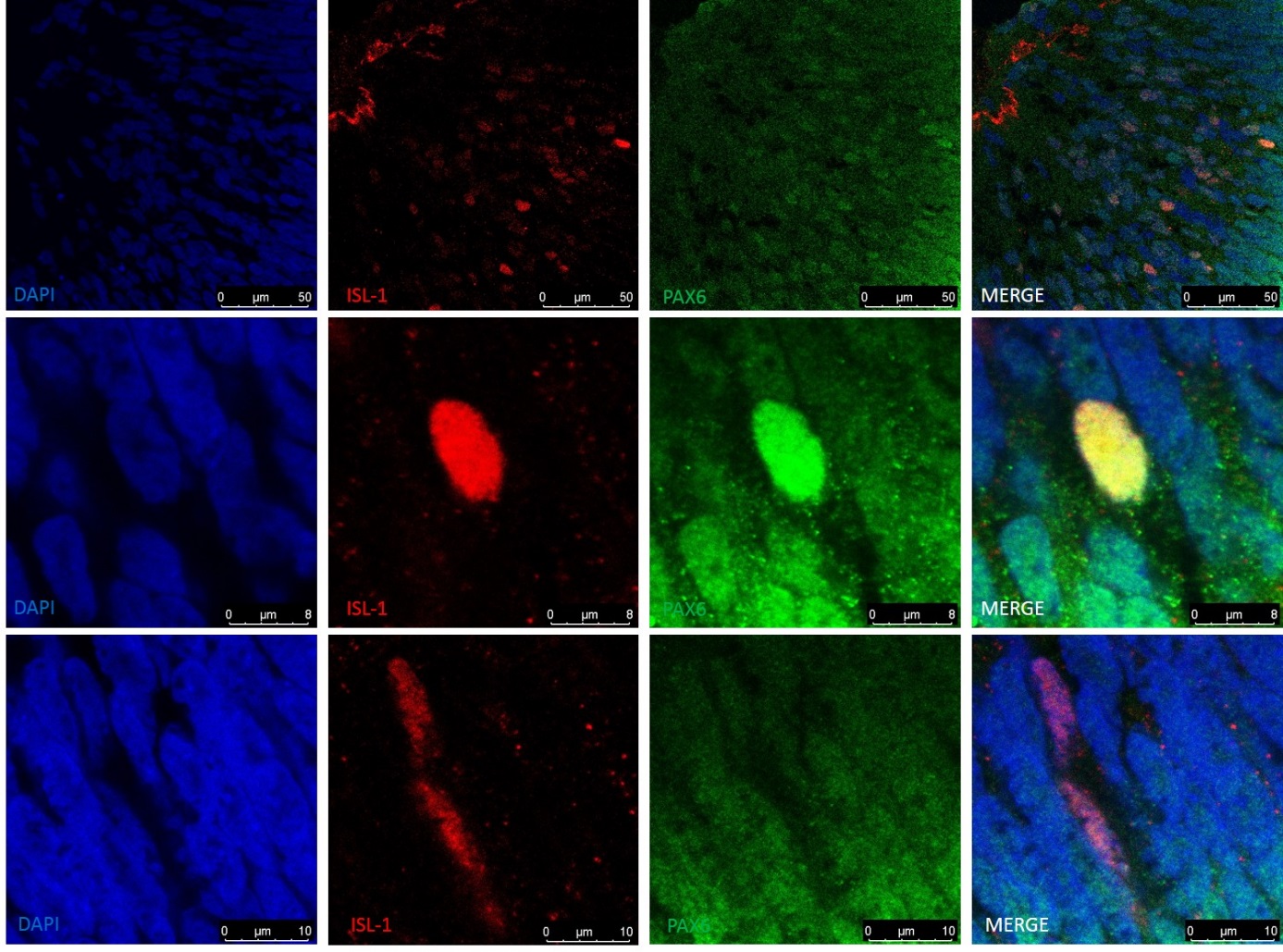


Figure S1

Figure S1. High magnification analysis of organoid slices showed that cells in the inner layer were both ISL1 and PAX6 positive, without the high overexpression of PAX6 on the outer edges of the organoid slices.
